# An Account of the Horn Distemper in Cattle, with Observations on That Disease

**Published:** 1786

**Authors:** Cotton Tufts


XI. *An Account of the Horn Diſtemper in Cattle, with Obſervations on that Diſeaſe. By the Hon.* Cotton Tufts, *M. D. F. A. A. and M. S. In a Letter to the Rev.* Joſeph Willard, *Cor. Sec. A. A.* — From the *Memoirs of the American Academy of Arts and Sciences,* Vol. I. 4to. Boſton, 1785.

BEASTS of the foreſt, guided by the dictates of nature, and uncontrolled by man in their food, air, exerciſe, and reſt, are ſeldom affected with any diſeaſe; whilſt, in almoſt all countries, the domeſtic kind, that are more immediately under the government of man, are ſubject to a variety.

Scarcely an inſtance in this country of reigning ſickneſs among tame or wild beaſts has been been noted by its hiſtorians; and it is within thirty years that we have heard much of epidemic diſeaſes among either.

About twenty-five years paſt an epidemic diſtemper prevailed among dogs, and occaſioned a great mortality. In 1768, horſes were generally affected with a diſorder of the head and throat, which proved fatal to many, and much injured the ſerviceableneſs of thoſe that ſurvived. About the year 1770, there were ſome inſtances of the *rabies canina*; happily but few dogs were affected, and but few perſons were bit; their rage principally fell upon ſwine. In 1771, a mortal diſtemper prevailed among foxes, and greatly reduced their numbers. About this time, or not long after, a diſtemper appeared among neat cattle, which deſtroyed many, and has continued to this day. The diſtempers that befel theſe ſeveral kinds of animals were ſaid not to have been known in the country before, more eſpecially that which has affected neat cattle, and which has generally been conſidered as a new diſeaſe: ſome, however, have ſuppoſed it to be the ſame which, from time to time, has made ravages in Europe, and more eſpecially in England. Whether it was ever known there is uncertain. It evidently evidently differs from thofe which Englifh writers have mentioned as proving fatal to their cattle. The compilers of the Complete Body of Hufbandry, re-publifhed in 1768, make no mention of this diforder, though they have treated largely of the diforders of horn cattle; thofe that have been more efpecially prevalent, and have proved mortal, they have defcribed under the names of *gargil*, *garget*, and *murrain*, and as attended with external fwellings, inflammations, eruptions, and contagion, and add, that "the murrain is the diftemper now, "and of many late years, fo fatal among the "horn-cattle." In 1757, Daniel Peter Layard, M. D. F. R. S. publifhed a particular account of the nature, caufes, and cure of the diftemper then among the horn-cattle in England. He confiders it as an eruptive difeafe, in the feveral ftages, progrefs, and effects of it, exactly the fame with the fmall-pox, and earneftly recommends inoculation. None of the external fwellings, eruptions, or contagion, characteriftic of thefe diforders, and to which fwine and fheep, as well as neat cattle, of all ages and kinds, are incident, are peculiar to this diforder. It is commonly called the horn diftemper. Cows are more efpecially fubject to

 it; it; oxen but feldom: bulls are faid to be exempt from it; alfo fteers and heifers, under three years of age. It is a difeafe that affects the internal fubftance of the horn, commonly called the pith, infenfibly waftes it, and leaves the horn hollow. The pith is a fpongy bone, whofe cells are filled with an unctuous matter: it is furnifhed with a great number of fmall blood veffels; is overfpread with a thin membrane, and appears to be united by future to the bones of the head, and is projected to a point. In a healthy beaft it fills up the cavity of the horn; the horn itfelf being a fheath or cafe giving firmnefs, and the whole ferving as a weapon of defence.

This fpongy bone, in the horn diftemper, is fometimes partly, and fometimes entirely, wafted. The horn lofes its natural heat, and a degree of coldnefs is evident upon handling it: when it is only in one horn, (which is often the cafe) a manifeft difference between the one and the other will be perceived, and in all cafes a want of natural heat will be apparent: wherever this is found, there is no room to doubt of the diforder's being prefent; yet it is feldom fufpected without a particular acquaintance with other fymptoms that commonly attend tend this diftemper; and, for want of knowing thefe, the farmer has often loft his cattle, not even fufpecting the evil.

Thefe fymptoms are a dulnefs in the countenance of the beaft, a fluggifhnefs in moving, a heavinefs of the eyes, a failure of appetite, an inclination to lay down, an averfion to rife, and, when accompanied with an inflammation of the brain, a giddinefs and frequent toffing of the head; befides thefe, the limbs are fometimes affected with ftiffnefs, like a rheumatifm, and in cows the milk often fails, the udder is hard, and in almoft all cafes there is a fudden wafting of the flefh.

As foon as the diforder is difcovered, an opening into the difeafed horn fhould be immediately made, which may be done with a twenty-penny-nail gimblet, in a part of the horn which might be fuppofed to be moft favourable for a difcharge: it is moft prudent to bore, at firft, two or three inches above the head; if it is found hollow, and the gimblet paffes through to the oppofite fide without refiftance, and no blood difcharges from the aperture, it may be beft to bore ftill lower, and as near the head as it fhall be judged that the hollownefs extends. This opening is a neceffary meafure, and often gives gives immediate relief. Care muſt be taken to keep it clear, as it is apt to be clogged by a thin fluid that gradually oozes out and fills up the paſſage. Some have practiſed ſawing off the horn; but, from the beſt obſervations, it does not ſucceed better than boring.

In autumn, 1774, on a farm not far from my houſe, I had four cows ſeized with this diſtemper in the ſpace of a fortnight: the firſt, an old cow, was affected with ſtiffneſs in her hind parts; her milk failed, her udder was hard and ſwelled, her eyes heavy, and her fleſh ſuddenly waſted. My tenant requeſted me to view her, upon an apprehenſion that ſhe had met with ſome hurt. At this time the diſorder was not much known among us. Fortunately a perſon fell in my way who had ſeen a ſimilar inſtance; and, upon relating the caſe, he ſuggeſted that it was the horn diſtemper, and upon examining her horns, one of them was found to be cold, and was immediately bored with a gimblet, which paſſed through to the oppoſite ſide without reſiſtance, and no diſcharge followed. Finding the horn hollow, I was led to think that the bones below were carious, and immediately made a mixture of rum and honey, with the addition of ſome tincture of myrrh and aloes, aloes, and syringed the horn; the injected liquor was soon discharged at her nose, tinged with blood: this was repeated several times, daily, and the injected liquor continued to run off through the nose for two or three days; at length it ceased to pass that way. Emollient fomentations were applied to her bag. These were the only applications. The cow, in a few days, shewed signs of recovery, but did not regain her flesh for several months. A second and third cow were taken ill; the disorder being early discovered, their horns bored, and syringed several times, they soon recovered. A fourth cow, about four years old, was observed in the morning to have the disorder: her horns were bored and syringed, her tail * cut for the purpose of bleeding, and from suspicion of defect in it: by nine o'clock she was scarce able to stand; at noon she was unable to rise, her head was very hot, her eyes dull, and she groaned as if in great pain; towards night she appeared as if near expiring, her eyes were

* Neat cattle are subject to a disorder, commonly called the tail sicknefs, which is a wasting of the bony substance of the tail; and if not cut off or dilated as far as the defect reaches, often proves fatal. It frequently accompanies the horn distemper.

unmoved unmoved on being touched, and the luſtre of them entirely gone; ſome degree of coldneſs, and a univerſal convulſion attended her. Under theſe circumſtances, I directed my tenant to take one ounce of powdered muſtard ſeed, to ſimmer it in a quart of milk, and add thereto one gill of molaſſes; the whole to be given immediately; afterwards to cover her over thick with ſtraw. This was ſoon done. In this ſtate ſhe was left in the evening. Before morning ſhe had eſcaped from her ſtraw, and was feeding in the field. She recovered without any farther application.

In the ſpring of 1779, another cow, of four years old, was ſeized. She was obſerved in the morning to refuſe her food; her eyes were heavy, ſhe hung down her head, and manifeſted an unhealthy countenance. The diſorder was ſuſpected; her horns examined; one of them felt cold, was bored, found hollow, and ſyringed: through the day ſhe was giddy, toſſing her head backward and forward; frequently groaned as if in great pain; and, upon rubbing her forehead, ſhewed ſigns of eaſe; her ſtrength was not much diminiſhed; her natural evacuations by ſtool and urine were free; however, ſhe died the next morning; and, according to the the information of my tenant, upon opening the body, the viſcera were all ſound; no mark of diſorder was ſeen there; but upon opening her head, the brain appeared of an unnatural colour, and, by his account, tending to a mortification.

From the number of cows ſeized with this diſtemper in the ſpace of a fortnight, as before mentioned, a ſuſpicion aroſe that the diſtemper was infectious; time, however, has ſhewn that it is not ſo, at leaſt in any great degree; for it frequently happens that, among many cattle herding together, one of them ſhall have the diſtemper, and the others remain in perfect health.

It appears from the firſt-recited caſe, that the injected liquor had a free paſſage from the horn to the noſe; yet, previous to the boring of the horn, there was no viſible diſcharge at the noſe of the waſted ſubſtance, or of any other matter, nor has there been in any other inſtance that I have heard of. As there appears no external diſcharge of the waſted bone, it muſt probably lodge in the cavities of the head, and, in proceſs of time, affect the brain; or the matter may be ſubtilized to a great degree, and be drawn into the circulation. It ſeems

ſurpriſing that ſo large a portion of bone as that which fills up the horn ſhould be deſtroyed, and the beaſt manifeſt no more complaints than are commonly obſerved; for the whole ſubſtance is generally loſt before the complaints riſe ſo high as to excite the notice of the farmer. To account for this, may it not be ſuppoſed that the mortification or diſſolution of the pith is attended with a degree of inſenſibility, and that the diſtreſs diſcovered does not exiſt in any great degree until the brain is in ſome meaſure affected, or the matter is abſorbed, and injures the habit in general?

Air bubbles are continually forming at the orifice through which the thin fluid oozes after the horn is bored. This indicates an internal fermentation, and it is not improbable that putrid matter of ſome kind or other may have given riſe to it. The matter may at firſt be formed on the perioſteum, and, entering into the interſtices of the bone, may diſſolve the oily ſubſtance, and form a fluid ſo putrid and corroſive as to diſſolve even the bone itſelf: upon this ſuppoſition, the air within becoming putrid and confined by the heat of the parts, will be largely expanded, from whence a great degree of compreſſion upon the ſurrounding parts parts muſt enſue: its effects at firſt may be ſmall—after a while great; at firſt producing no great diſtreſs—after a while ſome pain, but not ſufficient to produce ſuch uneaſy ſenſations as to be noticed; but when the bone is entirely waſted, and the putrid air much increaſed, and the compreſſion become great, the tender veſſels of the head muſt feel the force of it: the humours alſo may be highly acrimonious, and produce a general irritation. But from that ſenſible relief that an opening into the horn gives the beaſt, it is more than probable that the diſtreſs diſcovered ariſes from compreſſion, rather than from an effect produced on the blood and juices; for, in ſome inſtances, the beaſt is almoſt inſtantly relieved by making an opening in the horn.

The paſſing of liquors from the horn to the noſe, as in the caſe firſt mentioned, may perhaps be conſidered as an objection againſt the compreſſion ſuppoſed; but it is to be noted, that though there was a communication between the horn and the noſe in this caſe, yet it does not appear that there was any in divers other inſtances, or even in this after a few days.

From late obfervations I am led to conclude, that injections are in general unneceffary; that when the diftemper is early difcovered, no more is required than a proper opening into the horn, keeping it fufficiently clear for the admiffion of frefh air, the removal of the compreffion, and the difcharge of floating matter. But when the diftemper has communicated its effects to the brain, fo as to produce a high degree of inflammation, it is much to be doubted whether any method will fucceed.

